# Clinical Significance of Mean and Day-to-Day Variability of Home Blood Pressure in Chronic Kidney Disease: A Retrospective Cohort Study

**DOI:** 10.31662/jmaj.2025-0439

**Published:** 2025-12-05

**Authors:** Takeshi Tosaki, Daisuke Nakashima, Takaya Sasaki, Makoto Sagasaki, Yu Honda, Shinya Yokote, Nobuo Tsuboi, Takashi Yokoo

**Affiliations:** 1 Division of Nephrology and Hypertension, Department of Internal Medicine, The Jikei University School of Medicine, Tokyo, Japan; 2 Department of Nephrology, Kawaguchi Municipal Medical Center, Saitama, Japan

**Keywords:** chronic kidney disease, blood pressure, variability, home blood pressure monitoring, hypertension

## Abstract

**Introduction:**

Hypertension is a key risk factor for chronic kidney disease (CKD) progression. While mean blood pressure (BP) is well known to predict kidney outcomes, the role of BP variability (BPV)―especially day-to-day variability measured at home―in CKD remains unclear.

**Methods:**

In this retrospective cohort study of 150 patients with CKD, home systolic BP (SBP) was measured daily for up to 28 days. Mean SBP and day-to-day BPV (standard deviation of daily SBP) were calculated. Associations with the annual estimated glomerular filtration rate (eGFR) slope and a composite kidney endpoint (≥40% eGFR decline, kidney failure, or kidney-related death) were analyzed using linear mixed-effects models and Cox regression. The interaction between mean SBP and BPV on kidney outcomes was also evaluated.

**Results:**

The study population consisted of 150 patients (mean age, 66.1 years; 52% male) with a mean baseline eGFR of 43.2 mL/min/1.73 m^2^. Higher mean SBP was significantly associated with a steeper eGFR decline and increased risk of the composite kidney endpoint. In contrast, BPV was not independently associated with kidney outcomes. However, a significant interaction was observed, indicating that the detrimental effect of elevated mean SBP on kidney progression was amplified in patients with higher BPV.

**Conclusions:**

Mean home SBP was significantly associated with CKD progression. Although BPV alone was not independently related to outcomes, its interaction with mean SBP suggests that BP instability may exacerbate hypertension-related kidney damage. These findings highlight the clinical importance of both lowering and stabilizing BP in CKD management.

## Introduction

Hypertension is a well-established risk factor for the development of cardiovascular disease and chronic kidney disease (CKD) ^(1)^. Beyond absolute blood pressure levels, growing evidence indicates that blood pressure (BP) variability (BPV) may independently contribute to cardiovascular mortality and kidney-related outcomes ^(2), (3), (4)^. BPV is defined as fluctuations in blood pressure over various timeframes, including beat-to-beat (very short term), within 24 hours (short-term), day-to-day (mid-term), and visit-to-visit or monthly intervals (long-term).

In community-dwelling populations, several studies have shown that various BPV indices are associated with a decline in estimated glomerular filtration rate (eGFR) and an increased risk of kidney failure ^(3), (5)^. One study also demonstrated that day-to-day variability in home BP measurements is linked to incident CKD in a Japanese general population ^(6)^.

Among patients with prevalent CKD, prior studies evaluating the influence of short-term BPV using ambulatory BP monitoring or long-term BPV based on clinic visits have also shown potential associations with adverse kidney outcomes ^(7), (8)^. However, few studies have evaluated the association between day-to-day BPV assessed using home BP measurements and kidney dysfunction progression in this high-risk population. Therefore, in the present study, we aimed to investigate whether mean home BP and day-to-day BPV, calculated as the standard deviation (SD) of daily home systolic BP (SBP), are associated with kidney function decline and progression of kidney dysfunction in patients with CKD, and to evaluate whether day-to-day BPV modifies the relationship between mean home BP and kidney outcomes.

## Materials and Methods

### Study population

This retrospective cohort study was conducted at the Department of Nephrology, Kawaguchi Municipal Medical Center, Kawaguchi, Japan. In this department, nephrologists or outpatient clinic nurses routinely provide instructions on home BP measurement. Patients are encouraged to record their BP values in a diary and to bring the diary to each nephrology outpatient visit. Based on this routine clinical practice, we retrospectively collected data for the present study. This retrospective analysis was approved by the Institutional Review Board of Kawaguchi Municipal Medical Center (Approval code: 2025-19), which granted a waiver of consent. Patient data was included unless participants explicitly requested to opt out via public disclosure.

**Figure 1.**
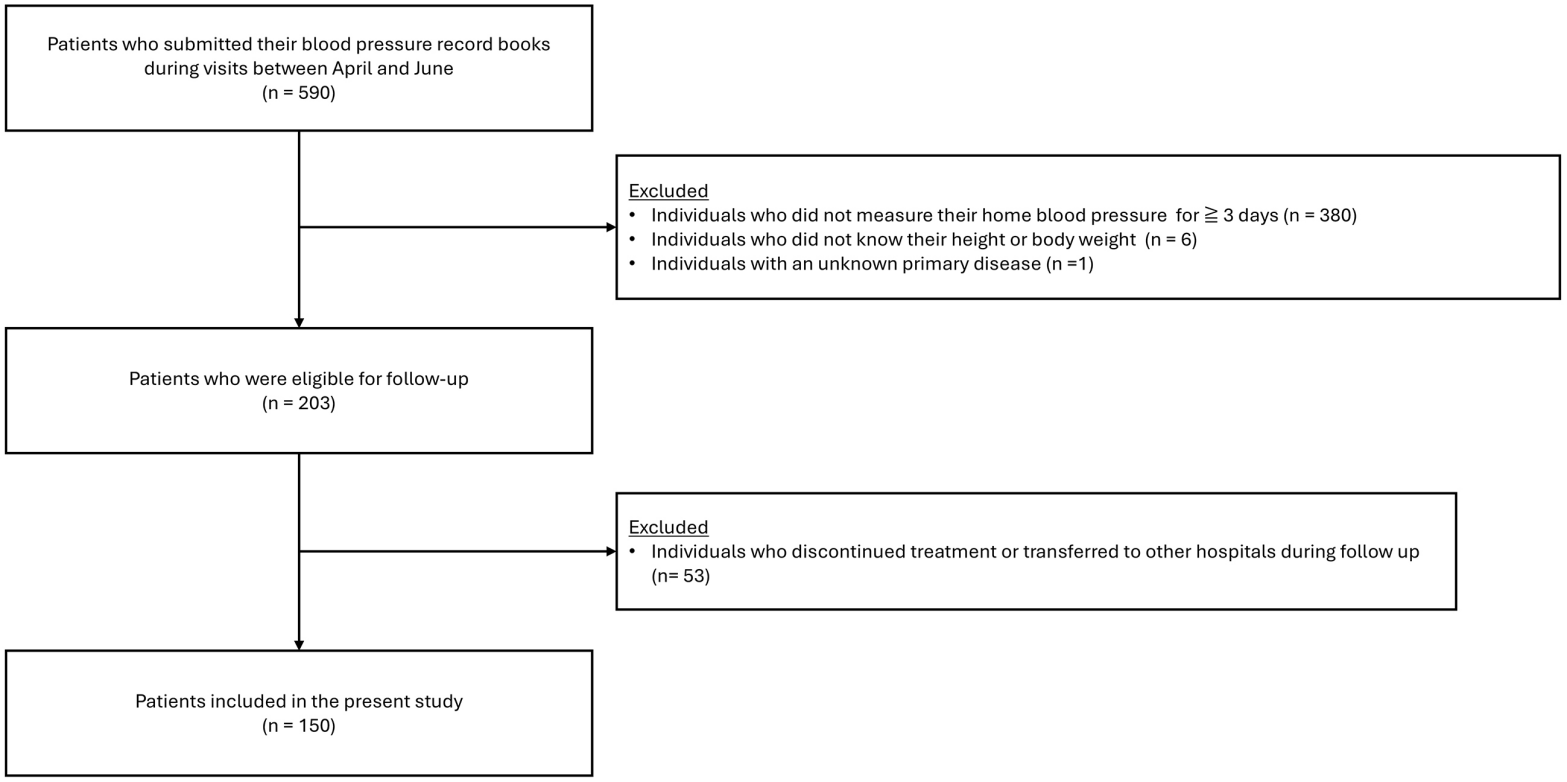
Flow diagram of patient selection.

The inclusion criteria were patients not receiving maintenance dialysis or kidney transplantation who submitted self-recorded home BP logs between April and June 2023. Patients were excluded if they had fewer than three days of home BP measurements, missing height or weight data, or an unknown primary kidney disease. As shown in Figure 1, a total of 590 patients were initially identified. Of these, 387 patients were excluded for the reasons listed above (insufficient BP data, n = 380; missing data, n = 6; unknown primary disease, n = 1), leaving 203 patients eligible for follow-up. During the two-year observation period, an additional 53 patients were excluded because they discontinued follow-up or were transferred to another facility. Thus, the final study population consisted of 150 patients. The primary diagnosis for kidney diseases was obtained from medical records.

### Exposures: BP parameters

As mentioned above, patients were routinely instructed during nephrology outpatient visits to measure their home BP three times each morning at one-minute intervals within one hour of waking, before breakfast, and before taking medications, after at least five minutes of seated rest. Participants used commercially available upper-arm automated BP monitors available in Japan; however, the device type was not standardized, and validation according to international protocols was not confirmed. Patients manually recorded their BP values in self-reported logs. The median duration of home BP monitoring was 27 days (range: 4-28 days). For each patient, the mean and SD of SBP were calculated from the daily average SBP values. Day-to-day BPV was defined as the SD of these daily SBP values. Patients were categorized into tertiles based on mean SBP and SD of SBP as follows: (i) mean SBP―tertile 1, <119.3 mmHg; tertile 2, 119.4-129.7 mmHg; tertile 3, ≥129.8 mmHg; and (ii) SD of SBP―tertile 1, <6.035 mmHg; tertile 2, 6.036-8.292 mmHg; tertile 3, ≥8.293 mmHg. In addition to SD, other BPV indices were evaluated, including the coefficient of variation (CV), calculated as SD divided by mean BP × 100, and variability independent of the mean (VIM), calculated as SD divided by the mean to the power of x, multiplied by the population mean to the power of x ^(9)^.

### Outcomes

The primary outcome was the annual eGFR slope, which has recently been established as a validated surrogate marker for kidney function decline and is known for its high sensitivity in detecting kidney dysfunction progression ^(10)^. The secondary outcome was a composite kidney endpoint defined as a ≥40% decline in eGFR, progression to kidney failure, or death related to kidney failure. Kidney failure was defined as initiation of kidney replacement therapy or an eGFR <15 mL/min/1.73 m^2^.

eGFR was evaluated every one to three months during routine outpatient visits throughout the follow-up period, and was calculated using the equation for the Japanese population ^(11)^. Serum creatinine was measured using an enzymatic method. Data for study outcomes and laboratory measures were collected until June 2025. Participants who did not reach the kidney endpoint were censored at their last outpatient visit during the follow-up period.

### Statistical analysis

The association between SBP parameters (e.g., mean SBP, SD of SBP) and eGFR slope was analyzed using linear mixed-effects models for repeated measures ^(12)^. The cumulative incidence of the composite kidney endpoint was estimated using the Kaplan-Meier method, and differences among exposure levels were assessed using the log-rank test. Hazard ratios for the composite kidney endpoint according to tertiles of SBP were calculated using the Cox proportional hazards model. Multivariable-adjusted models were adjusted for age, sex, urinary protein-to-creatinine ratio (log-transformed), use of renin-angiotensin system inhibitors, and use of sodium-glucose cotransporter 2 inhibitors. The model also tested for trends across the SBP tertiles using an ordinal variable. Interactions between subgroups were tested by adding multiplicative terms to the model. A competing risk analysis was also conducted for all-cause death using the Fine and Gray method. Receiver operating characteristic (ROC) curve analyses were performed to evaluate the predictive ability of mean SBP, SD, CV, and VIM for the composite kidney endpoint. Sensitivity analyses were performed for the CV of SBP and VIM of SBP as BPV indices. In subgroup analyses, interactions between the mean SBP and SD of SBP were tested for associations with kidney outcomes (annual eGFR slope and composite endpoint).

All statistical tests were two-sided, and p-values <0.05 were considered statistically significant. Analyses were conducted using SAS software, version 9.4 (SAS Institute, Cary, NC, USA).

## Results

### Baseline characteristics

The study population consisted of 150 patients with a mean age of 66.1 years; 52% were male. The mean home SBP was 125.5 mmHg, and the mean SD of home SBP was 7.85 mmHg. Baseline characteristics stratified by tertiles of mean home SBP and SD of home SBP are presented in Table 1. Higher mean SBP was significantly associated with older age, higher mean diastolic BP (DBP), and lower eGFR. Similarly, higher SD of SBP was associated with older age, higher mean SBP and DBP, and lower eGFR.

**Table 1.** Baseline Characteristics.

Variables	Overall	Tertiles of mean home SBP, mmHg			p for trend	Tertiles of SD levels of home SBP, mmHg			p for trend
		Tertile 1, <119.3	Tertile 2, 119.4-129.7	Tertile 3, ≥129.8		Tertile 1, <6.035	Tertile 2, 6.036-8.292	Tertile 3, ≥8.293	
	n = 150	n = 50	n = 50	n = 50		n = 50	n = 50	n = 50	
Age, years	66.1 (16.2)	58.9 (17.1)	69.1 (14.1)	70.4 (15.1)	<0.001	60.5 (16.6)	64.2 (17.3)	73.6 (11.6)	<0.001
Male, n (%)	78 (52)	23 (46)	26 (52)	29 (58)	0.23	26 (52)	27 (54)	25 (50)	0.84
Mean home SBP, mmHg	125.5 (12.5)	112.5 (6.2)	125.1 (2.9)	138.8 (8.5)	<0.001	119.8 (11.2)	125.2 (10.4)	131.4 (13.1)	<0.001
Mean home DBP, mmHg	75.8 (8.6)	72.0 (5.1)	75.7 (8.5)	79.7 (10.2)	<0.001	75.2 (6.8)	76.0 (8.9)	76.2 (10.3)	0.83
Antihypertensive medication use, n (%)	118(78.7)	36 (72)	41 (82)	41 (82)	0.22	40 (80)	39 (78)	39 (78)	0.81
Calcium channel blocker	73 (48.7)	16 (32.0)	26 (52.0)	31 (62.0)	0.003	23 (46.0)	24 (48.0)	26 (52.0)	0.55
Renin-angiotensin system inhibitor	97 (64.7)	31 (62.0)	35 (70.0)	31 (62.0)	1.00	35 (70.0)	29 (58.0)	33 (66.0)	0.68
Angiotensin receptor-neprilysin inhibitor	3 (2.0)	0 (0.0)	0 (0.0)	3 (6.0)	0.032	1 (2.0)	2 (4.0)	0 (0.0)	0.48
Mineralocorticoid receptor antagonist	11 (7.3)	2 (4.0)	4 (8.0)	5 (10.0)	0.45	2 (4.0)	3 (6.0)	6 (12.0)	0.12
Beta-blocker	15 (10.0)	1 (2.0)	6 (12.0)	8 (16.0)	0.020	4 (8.0)	5 (10.0)	6 (12.0)	0.51
Alpha-blocker	11 (7.3)	1 (2.0)	2 (4.0)	8 (16.0)	0.007	3 (6.0)	4 (8.0)	4 (8.0)	0.70
Thiazide diuretic	15 (10.0)	2 (4.0)	4 (8.0)	9 (18.0)	0.020	4 (8.0)	7 (14.0)	4 (8.0)	1.00
Sodium-glucose cotransporter-2 inhibitor	23 (15.3)	7 (14.0)	11 (22.0)	5 (10.0)	0.58	5 (10.0)	8 (16.0)	10 (20.0)	0.17
Primary kidney disease, n (%)*					0.47*				0.22*
Nephrosclerosis	48 (32)	16 (32)	17 (34)	15 (30)	NA	21 (42)	14 (28)	13 (26)	NA
Diabetic kidney disease	10 (6.7)	1 (2)	3 (6)	6 (12)	NA	0 (0)	4 (8)	6 (16)	NA
Chronic glomerulonephritis	84 (56)	31 (62)	28 (56)	25 (50)	NA	27 (54)	29 (58)	28 (56)	NA
Autosomal dominant polycystic kidney disease	8 (5.3)	2 (4)	2 (4)	4 (8)	NA	2 (4)	3 (6)	3 (6)	NA
Diabetes, n (%)	38 (25.3)	11 (22)	10 (20)	17 (34)	0.17	8 (16)	11 (22)	19 (38)	0.011
Serum LDL cholesterol, mmol/L	2.67 (0.71)	2.70 (0.68)	2.58 (0.59)	2.73 (0.83)	0.55	2.66 (0.66)	2.77 (0.67)	2.58 (0.79)	0.39
Use of lipid-modifying medication, n (%)	48 (32)	11 (22)	20 (40)	17 (34)	0.20	16 (32)	19 (38)	13 (26)	0.52
Hypercholesterolemia, %	72 (48)	20 (40)	27 (54)	25 (50)	0.32	26 (52)	25 (50)	21 (42)	0.32
Body mass index, kg/m^2^	23.3 (4.5)	23.0 (4.2)	23.6 (5.2)	23.8 (4.3)	0.81	23.9 (4.7)	23.4 (4.4)	22.7 (4.5)	0.40
Serum uric acid, mg/dL	5.5 (1.2)	5.9 (1.4)	5.7 (1.3)	6.0 (1.1)	0.15	5.6 (1.3)	5.7 (1.1)	6.1 (1.3)	0.19
Estimated glomerular filtration rate, mL/min per 1.73 m^2^	43.2 (20.7)	48.3 (18.9)	43.1 (20.8)	38.3 (21.4)	0.054	47.8 (21.8)	47.3 (23.3)	34.6 (12.9)	0.001
Urine protein, g/gCr	0.83 (1.39)	0.49 (0.79)	0.69 (1.04)	1.30 (1.97)	0.009	0.66 (1.05)	0.67 (1.16)	1.15 (1.80)	0.13
Current smoking habits, n (%)	15 (10.0)	3 (6)	3 (6)	9 (18)	0.05	3 (6)	7 (14)	5 (10)	0.51
Current drinker, n (%)	49 (32.7)	16 (32)	18 (36)	15 (30)	0.83	14 (28)	20 (40)	15 (30)	0.83

DBP: diastolic blood pressure; LDL: low-density lipoprotein; NA: not applicable; SBP: systolic blood pressure; SD: standard deviation. *p-values were calculated by the chi-square test.

### Association with annual eGFR decline

**Figure 2.**
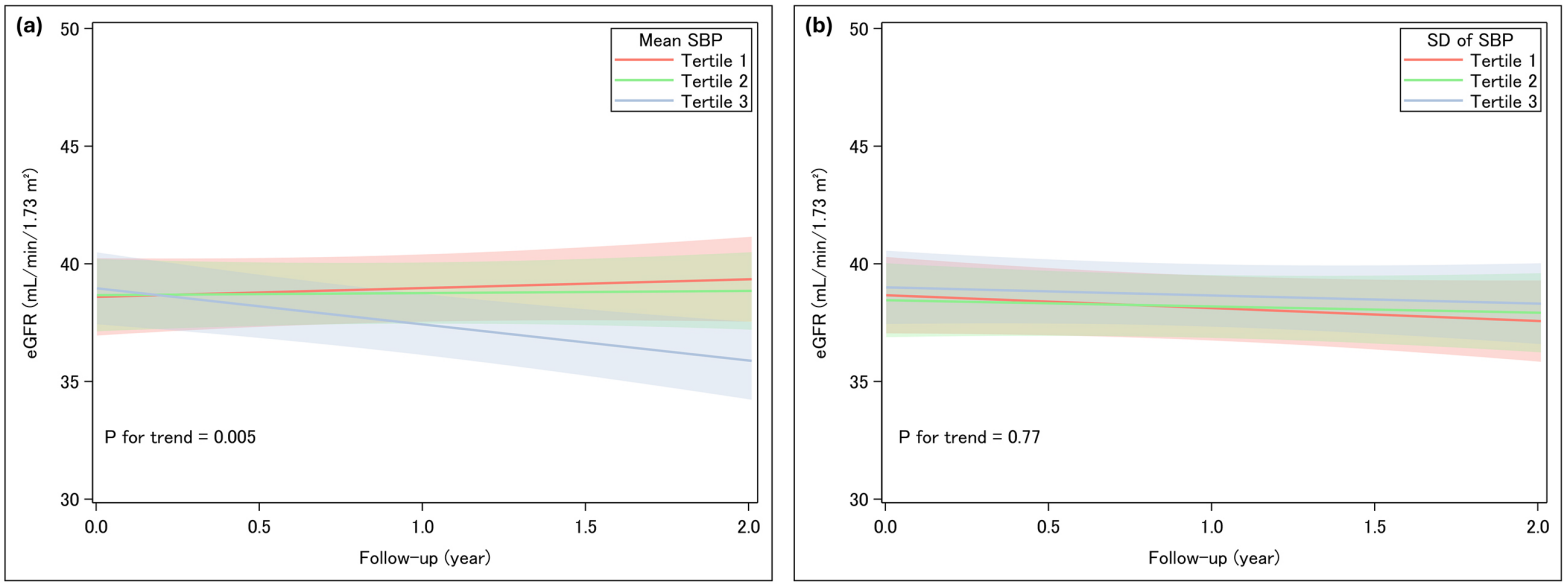
Longitudinal changes in eGFR stratified by systolic blood pressure SBP parameters. (a) Mean SBP and (b) SD of SBP were categorized into tertiles. Individual lines represent the estimated eGFR trajectory over a two-year follow-up for each tertile, based on linear mixed-effects models for repeated measures. Shaded areas indicate 95% confidence intervals. eGFR: estimated glomerular filtration rate; SBP: systolic blood pressure; SD: standard deviation.

The multivariable-adjusted mixed-effects model for repeated measures revealed that higher mean SBP was significantly associated with a steeper decline in annual eGFR slope (*p* for trend = 0.005; Figure 2a). The annual eGFR slopes (mL/min/1.73 m^2^/year) with 95% confidence intervals for each tertile of mean SBP were +0.37 (-0.59, +1.34), -0.09 (-0.83, +1.01), and -1.54 (-2.46, -0.62), respectively. In contrast, BPV indices of SBP (SD, CV, and VIM) showed no significant associations with eGFR slope (Figure 2b, Figure S1). Similarly, mean DBP was associated with kidney outcomes, but DBP variability was not (Figure S2).

### Composite kidney endpoint

**Figure 3.**
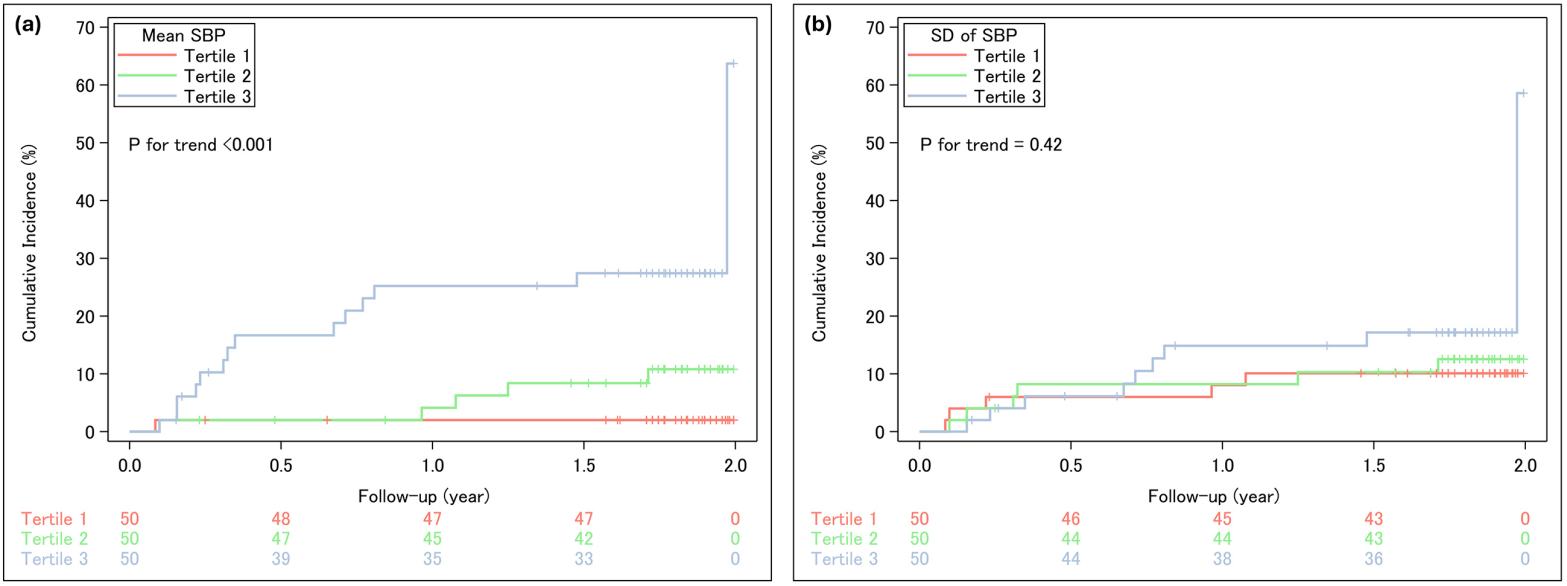
Incidence of the kidney composite endpoint stratified by SBP parameters. (a) Mean SBP and (b) SD of SBP were categorized into tertiles. Individual lines represent the incidence of the composite kidney endpoint over a two-year follow-up for each tertile, based on Kaplan-Meier analysis. The composite kidney endpoint was defined as a ≥40% decline in estimated glomerular filtration rate, progression to kidney failure, or death from kidney failure. eGFR: estimated glomerular filtration rate; SBP: systolic blood pressure; SD: standard deviation.

During the two-year follow-up period, 20 patients reached the composite kidney endpoint, defined as a ≥40% decline in eGFR, eGFR <15 mL/min/1.73 m^2^, or initiation of kidney replacement therapy. Kaplan-Meier curves showed that patients with higher mean SBP had a significantly greater risk of kidney events (p for trend <0.001; Figure 3a). BP variability indices were not significantly associated with this composite endpoint (Figure 3b; Figure S3).

**Figure 4.**
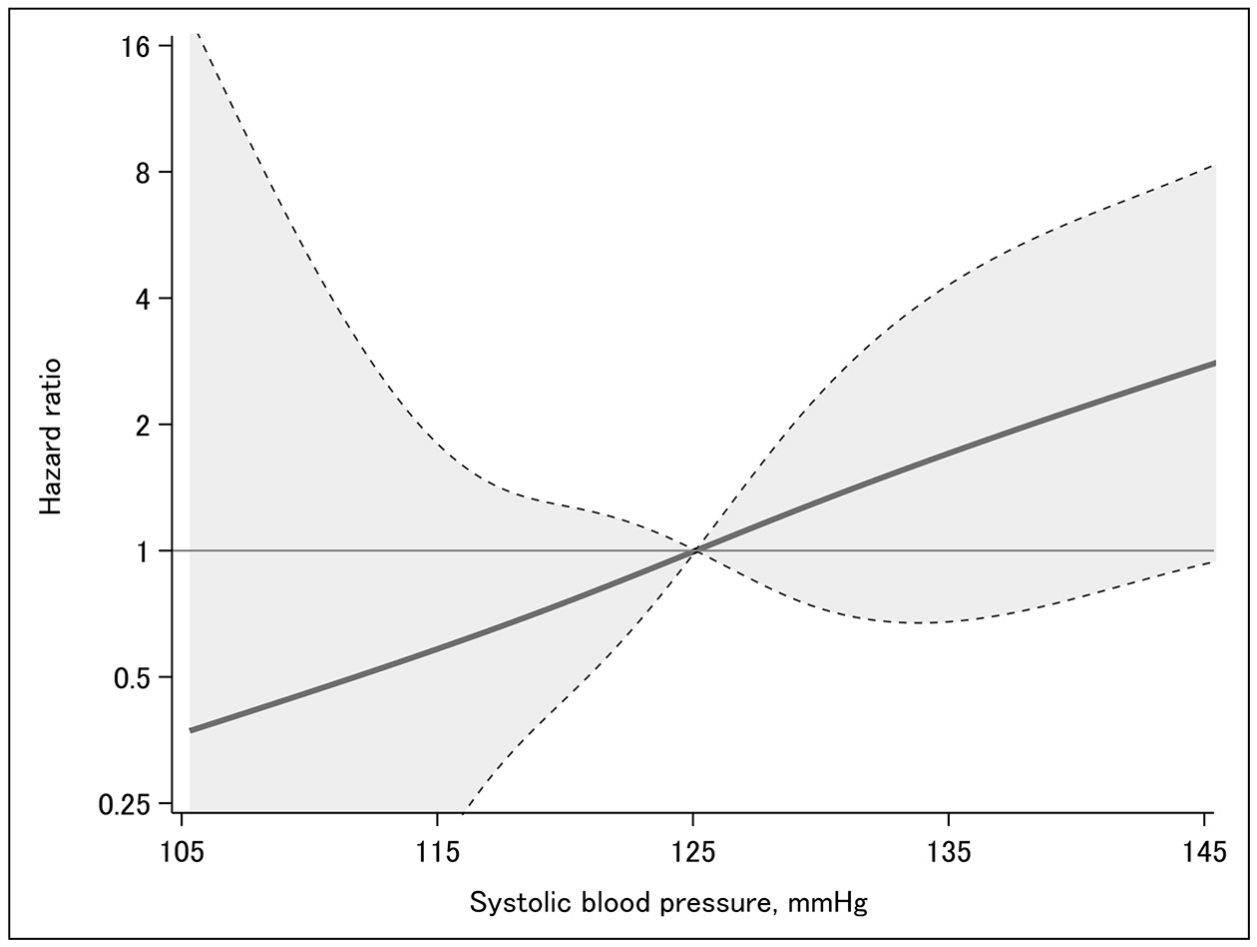
Multivariable-adjusted cubic spline curve showing the association between mean SBP and the composite kidney endpoint. A restricted cubic spline curve with 95% confidence intervals (gray shaded areas) illustrates the cumulative incidence of the composite kidney endpoint over the two-year follow-up. The composite kidney endpoint was defined as a ≥40% decline in estimated glomerular filtration rate, progression to kidney failure, or death from kidney failure. Results were adjusted for age, sex, urinary protein-to-creatinine ratio (log-transformed), use of renin-angiotensin system inhibitors, and use of sodium-glucose cotransporter two inhibitors. SBP: systolic blood pressure.

In the multivariable-adjusted Cox proportional hazards model and the multivariable-adjusted Fine-Gray model with death as a competing risk, mean SBP showed a significant positive association with the composite kidney endpoint (Table 2). Moreover, a cubic spline analysis revealed a linear association between mean SBP and the composite kidney endpoint (Figure 4).

**Table 2.** Hazard Ratios for the Composite Kidney Endpoint by Tertiles of Mean Systolic Blood Pressure.

Tertile of mean home SBP, mmHg	At risk, n	Events, n	Competing events (deaths), n	Crude		Multivariable-adjusted	
				HR (95% CI)	p for trend	HR (95% CI)	p for trend
*Cox proportional hazards model*							
Tertile 1, <119.3	50	1	NA	1 (reference)	<0.001	1 (reference)	0.016
Tertile 2, 119.4-129.7	50	5	NA	5.3 (0.6-45.2)		4.2 (0.5-37.9)	
Tertile 3, ≥129.8	50	14	NA	17.4 (2.3-132.6)		9.2 (1.1-77.6)	
*Fine-Gray subdistribution hazards model*							
Tertile 1, <119.3	50	1	1	1 (reference)	<0.001	1 (reference)	0.005
Tertile 2, 119.4-129.7	50	5	1	5.3 (0.6-46.0)		4.3 (0.6-34.4)	
Tertile 3, ≥129.8	50	14	2	16.5 (2.1-130.4)		9.1 (1.4-59.6)	

Results were adjusted for age, sex, urinary protein-to-creatinine ratio (log-transformed), use of renin-angiotensin system inhibitors, and use of sodium-glucose cotransporter 2 inhibitors. CI: confidence interval; HR: hazard ratio; NA: not applicable; SBP: systolic blood pressure.

**Figure 5.**
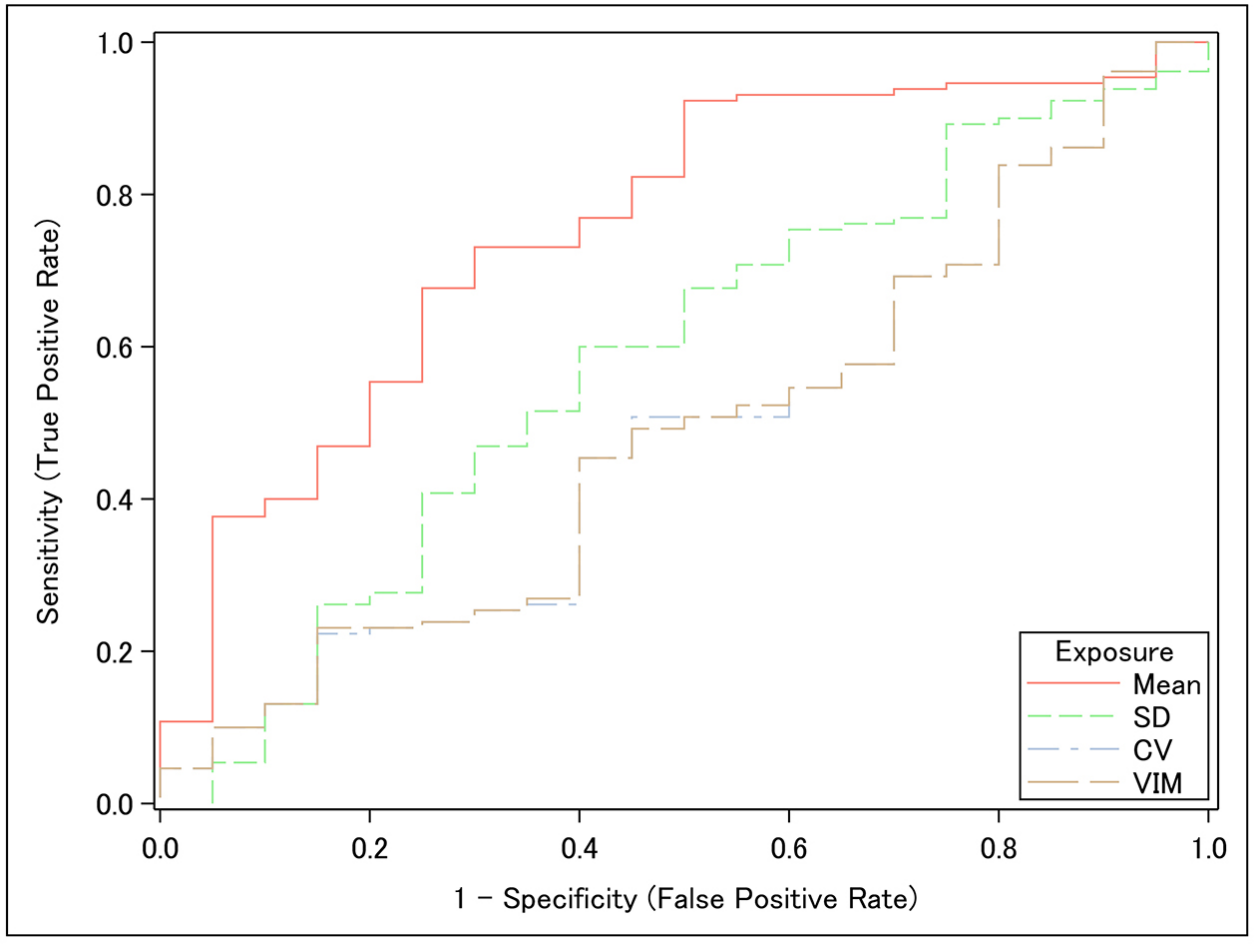
ROC curves illustrating the predictive utility of SBP parameters for the composite kidney endpoint. ROC curves were generated for mean SBP, SD of SBP, CV of SBP, and VIM of SBP. The composite kidney endpoint was defined as a ≥40% decline in estimated glomerular filtration rate, progression to kidney failure, or death from kidney failure. CV: coefficient of variation; ROC: receiver operating characteristic; SBP: systolic blood pressure; SD: standard deviation; VIM: variability independent of the mean.

A ROC curve analysis was performed to evaluate the discriminative ability of SBP indices. The discriminative ability of mean SBP for the composite kidney endpoint was superior to that of BP variability indices (i.e., SD, CV, and VIM) (Figure 5).

### Subgroup analysis: interaction of mean SBP with BPV

**Figure 6.**
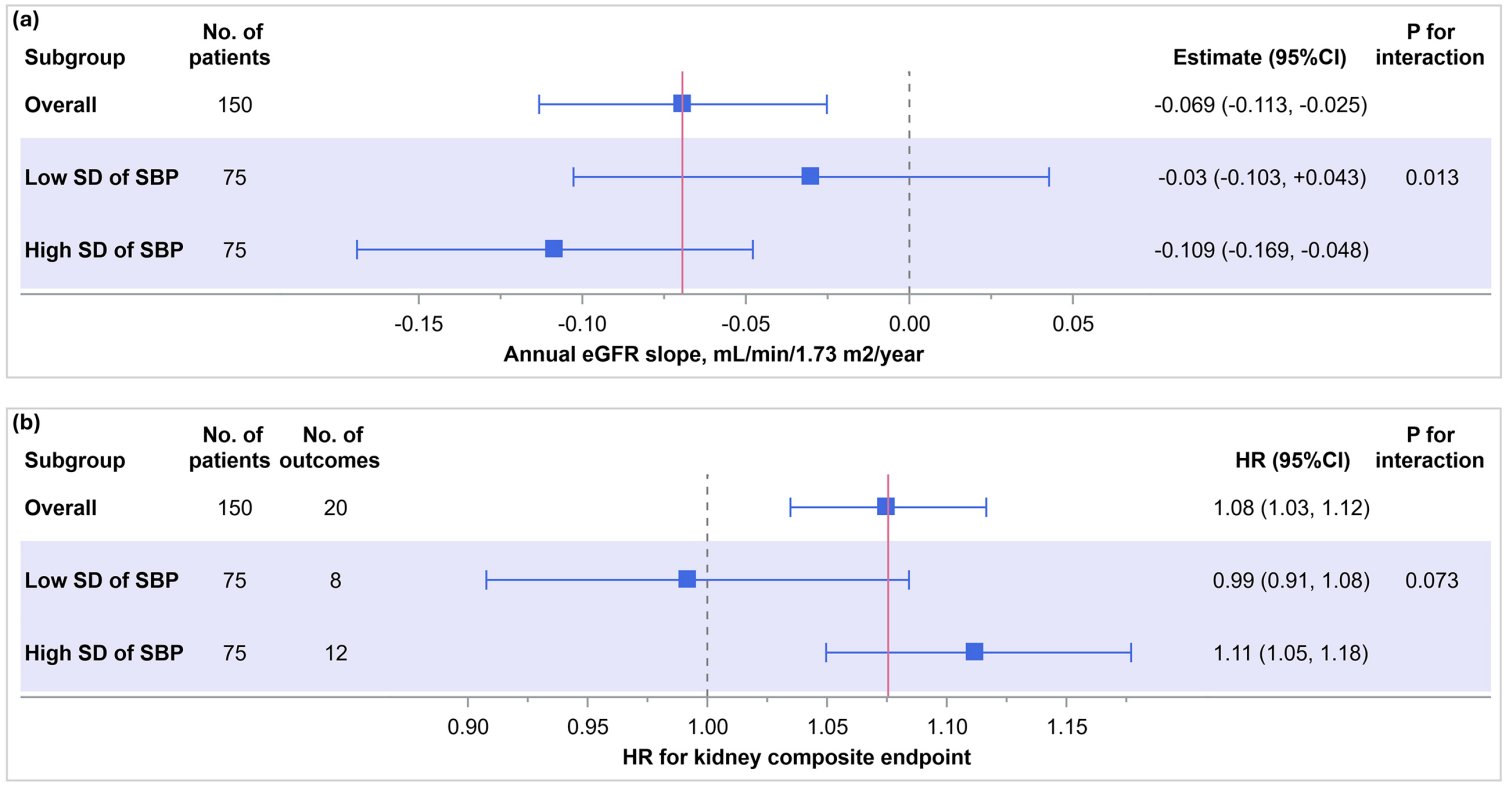
Association of mean SBP with kidney outcomes stratified by SD of SBP. The associations between mean SBP and (a) the annual eGFR slope and (b) the incidence of the composite kidney endpoint were stratified by the median SD of SBP. For eGFR slope (a): linear mixed-effects models were used; for the composite outcome (b): Cox proportional hazards models were applied. eGFR: estimated glomerular filtration rate; SBP: systolic blood pressure; SD: standard deviation.

As shown in Figure 6, the associations between mean SBP and kidney outcomes were modified by SBP variability. In the low SD of SBP group, the annual eGFR slope (mL/min/1.73 m^2^/year) associated with mean SBP was -0.03 (95% CI: -0.103 to +0.043), whereas in the high SD group, the slope was steeper at -0.109 (95% CI: -0.169 to -0.048), with a significant interaction (*p* for interaction = 0.013). Similarly, the hazard ratio for the composite kidney endpoint per unit increase in mean SBP was 0.99 (95% CI: 0.91 to 1.08) in the low SD group and 1.11 (95% CI: 1.05 to 1.18) in the high SD group, with a marginal interaction (*p* = 0.073).

## Discussion

In this study of patients with CKD, higher mean SBP was significantly associated with a steeper decline in eGFR and a higher risk of kidney disease progression, and it showed good predictive ability for progression in ROC analysis. In contrast, the BPV indices SD, CV, and VIM were not independently associated with these outcomes. Notably, we observed a significant interaction indicating that the adverse effect of elevated mean SBP on kidney outcomes was more pronounced among patients with greater BPV.

While the association between higher mean BP and kidney disease progression is well-established ^(13), (14), (15)^, the role of BPV remains less clear. Several studies have examined visit-to-visit BPV in patients with CKD, but their findings have been inconsistent ^(8), (16), (17), (18), (19), (20)^. Our study contributes to this area by focusing on day-to-day BPV assessed through home BP monitoring, which may more accurately capture short-term hemodynamic stress. To date, only one previous study, by Okada et al. ^(21)^, has evaluated this type of BPV in CKD patients, and it similarly found no association with kidney outcomes. However, their study did not assess potential effect modification by BPV. In our analysis, while day-to-day BPV was not independently associated with eGFR decline or kidney failure, we found that elevated mean BP had a more pronounced adverse effect in individuals with higher BPV. These findings suggest that mean BP remains a dominant predictor of CKD progression. Although BPV itself was not an independent risk factor, its interaction with mean BP indicates that patients with unstable BP may be more vulnerable to the harmful effects of hypertension. This underscores the importance of not only achieving but also maintaining stable BP control in CKD management. Clinicians should therefore consider both average BP levels and day-to-day variability when adjusting antihypertensive treatment, particularly in patients with fluctuating home BP readings.

The synergistic effect of elevated mean BP and high BPV may be explained by increased hemodynamic stress and endothelial dysfunction. In animal models, Eto et al. ^(22)^ showed that increased BPV impairs endothelial function by inhibiting nitric oxide production and promoting neointimal formation, independently of mean BP levels, while Nakano et al. ^(23)^ reported a negative association between visit-to-visit BPV and flow-mediated dilation in CKD patients, suggesting BPV-related endothelial injury. In CKD, impaired autoregulatory capacity of the kidney increases susceptibility to glomerular damage even from moderate BP elevations ^(24)^, and BPV may further exacerbate this glomerular vulnerability through endothelial stress. Additionally, increased day-to-day BPV has been linked to arterial stiffness in the general population ^(25)^, and experimental studies in hypertensive rats have shown that higher BPV alone can worsen arteriosclerosis in the kidney microvasculature ^(26)^. These findings suggest that combined exposure to elevated mean BP and BPV may amplify vascular injury and promote CKD progression. Moreover, elevated BP levels and variability may indicate poor medication adherence, which could also contribute to adverse kidney outcomes ^(27), (28)^.

Several limitations of this study should be acknowledged. First, its observational design precludes causal inference between BP measures and kidney outcomes. Second, the relatively small sample size reduced statistical power and may have prevented the detection of an independent association between BPV and kidney outcomes. Third, the subgroup analyses were exploratory and hypothesis-generating, warranting confirmation in larger prospective studies. Fourth, potential confounding from unmeasured variables, such as medication adherence, may partly explain the lack of an independent association between BPV and kidney outcomes. Fifth, variability in BP measurement may have arisen from non-standardized devices, unassessed adherence, and the self-reported nature of home BP monitoring. Finally, excluding participants with fewer than three days of measurements may have introduced selection bias toward more adherent or health-conscious individuals, or those with more severe disease.

### Conclusions

Mean home SBP is a robust and clinically relevant predictor of kidney disease progression in patients with CKD. While BPV was not an independent predictor, its interaction with mean BP suggests that BP instability may amplify the harmful effects of hypertension. Future studies should explore interventions targeting not only BP reduction but also BP stabilization to optimize kidney and cardiovascular outcomes in this population.

## Article Information

### Acknowledgments

We thank the patients who participated in this study. We are also grateful to the medical staff at Kawaguchi Municipal Medical Center for their valuable contributions to data collection and patient care.

### Author Contributions

Conceptualization & Design; Data Curation; Formal Analysis & Visualization; Investigation; Manuscript Drafting; Review & Editing: Takeshi Tosaki, Daisuke Nakashima, and Takaya Sasaki. Investigation; Review & Editing: Makoto Sagasaki and Yu Honda. Supervision; Review & Editing: Shinya Yokote, Nobuo Tsuboi, and Takashi Yokoo. Contributed equally to this work: Takeshi Tosaki and Daisuke Nakashima.

### Conflicts of Interest

None

### IRB Approval Code and Name of the Institution

This study was approved by the Institutional Review Board of Kawaguchi Municipal Medical Center (Approval code: 2025-19).

## Supplement

Supplementary Material
