# Supplementary material for: Clinical Significance of Mean and Day-to-Day Variability of Home Blood Pressure in Chronic Kidney Disease: A Retrospective Cohort Study
Source: JMA J. 2025 Dec 5;9(1):331–9. doi: 10.31662/jmaj.2025-0439 (PMC12889104; doi:10.31662/jmaj.2025-0439)
Supplement: Supplementary Material [file 2433-3298-9-1-0331-s001.pdf]

Supplementary Materials

<u>Table of contents</u>

Supplementary Fig. S1. Longitudinal changes in estimated glomerular filtration rate (eGFR) stratified by systolic blood pressure (SBP) parameters.
Supplementary Fig. S2. Longitudinal changes in estimated glomerular filtration rate (eGFR) stratified by diastolic blood pressure (DBP) parameters.
Supplementary Fig. S3. Incidence of the composite kidney endpoint stratified by systolic blood pressure (SBP) parameters.

Supplementary Fig. S1. Longitudinal changes in estimated glomerular filtration rate (eGFR) stratified by systolic blood pressure (SBP) parameters.

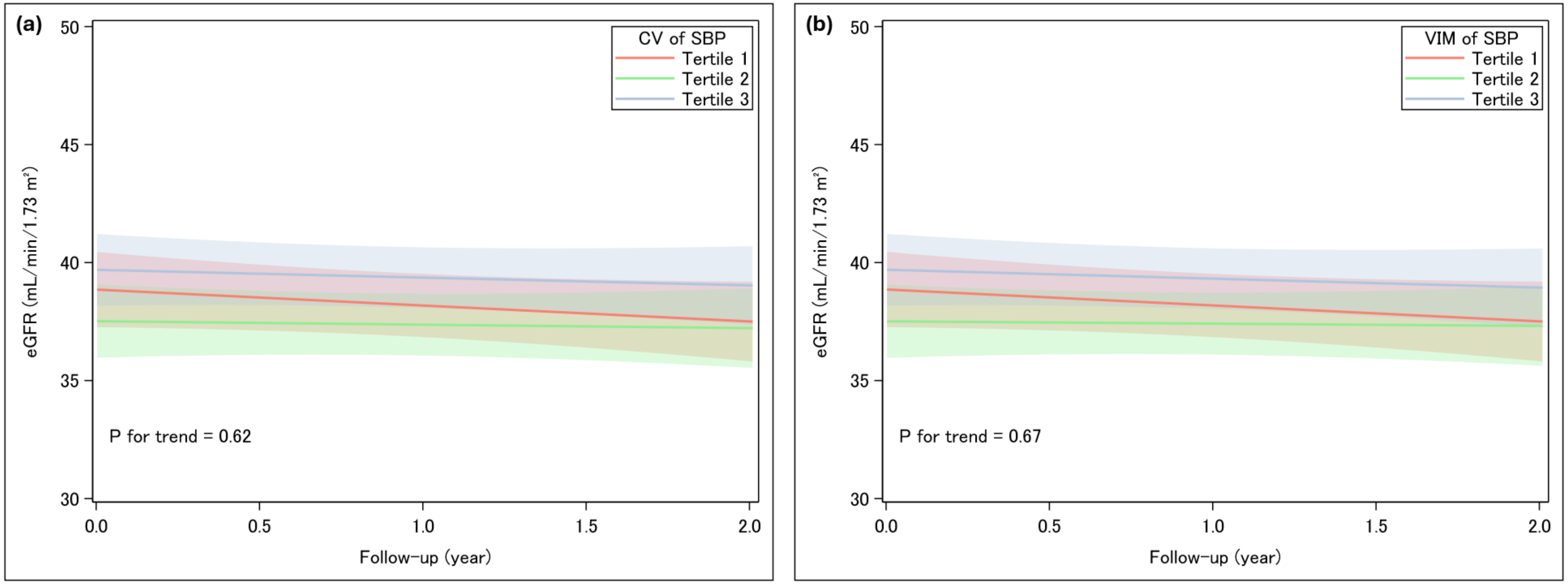

(a) Coefficient of variation (CV) of SBP and (b) Variation of independent of the mean (VIM) of SBP were categorized into tertiles. Each line represents the estimated eGFR trajectory over a 2-year follow-up according to tertiles, based on linear mixed-effects models for repeated measures. Shaded areas indicate 95% confidence intervals.

Supplementary Fig. S2. Longitudinal changes in estimated glomerular filtration rate (eGFR) stratified by diastolic blood pressure (DBP) parameters.

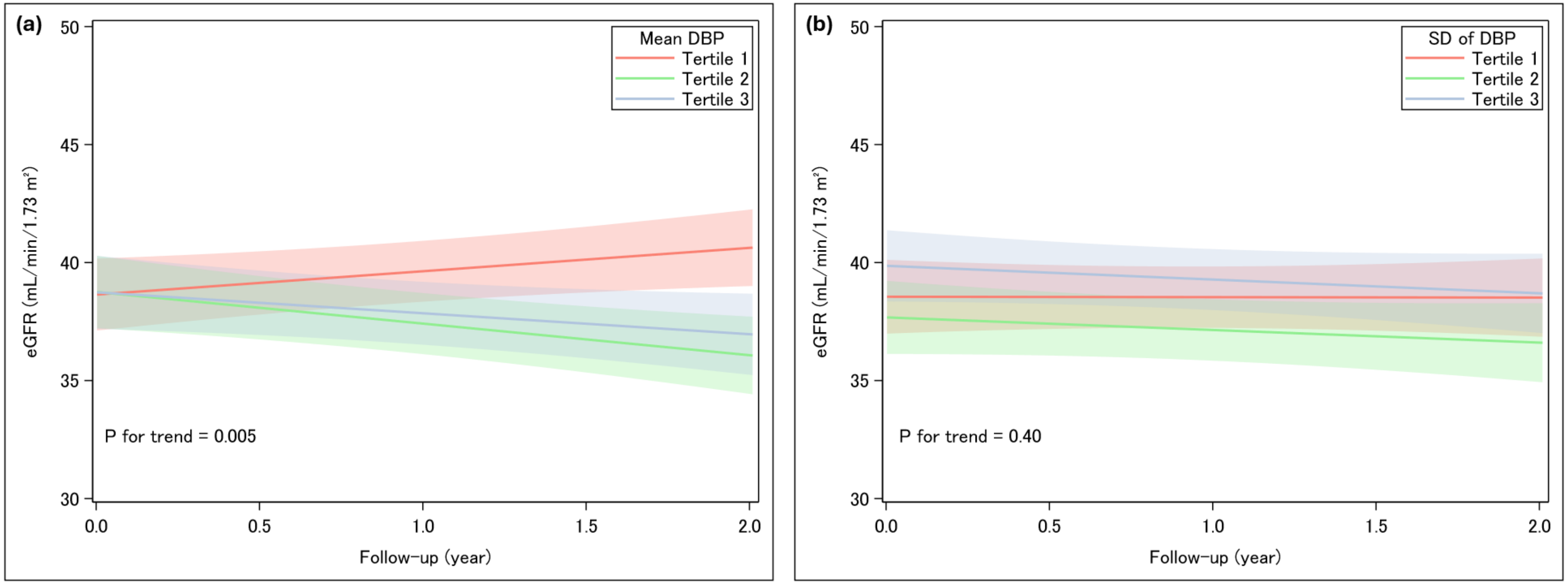

(a) Mean DBP and (b) Standard deviation (SD) of DBP were categorized into tertiles. Each line represents the estimated eGFR trajectory over a 2-year follow-up according to tertiles, based on linear mixed-effects models for repeated measures. Shaded areas indicate 95% confidence intervals.

Supplementary Fig. S3. Development of kidney composite outcomes stratified by systolic blood pressure (SBP) parameters.

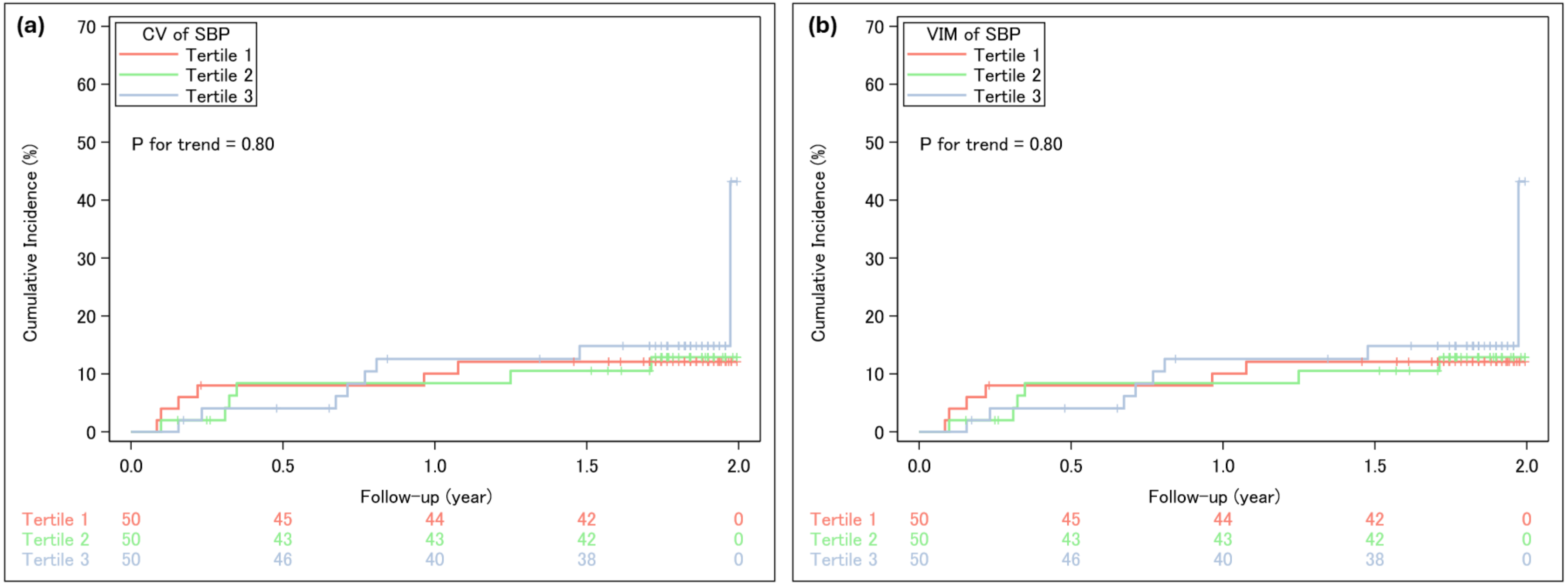

(a) Coefficient of variation (CV) of SBP and (b) Variation of independent of the mean (VIM) of SBP were categorized into tertiles. Each line represents incident kidney composite outcomes over a 2-year follow-up according to tertiles, based on Kaplan-Meier analysis. Kidney composite outcomes were defined as a ≥40% decline in estimated glomerular filtration rate, progression to kidney failure, or death from kidney failure.
